# Age and liver graft: a systematic review with meta-regression

**DOI:** 10.1007/s13304-023-01641-1

**Published:** 2023-09-11

**Authors:** Ilaria Neri, Marco Maria Pascale, Giuseppe Bianco, Francesco Frongillo, Salvatore Agnes, Francesco Giovinazzo

**Affiliations:** General Surgery and Liver Transplant Unit, Fondazione Policlinico Universitario Agostino Gemelli IRCCS, Rome, Italy

**Keywords:** Liver transplantation, Extended criteria donors, Elderly donors

## Abstract

Increasing organ shortage results in extended criteria donors (ECD) being used to face the growing demand for liver grafts. The demographic change leads to greater use of elderly donors for liver transplantation, historically considered marginal donors. Age is still considered amongst ECD in liver transplantation as it could affect transplant outcomes. However, what is the cutoff for donor age is still unclear and debated. A search of PubMed, Scopus and Cochrane Library was performed. The primary outcome was 1-year graft survival (GS). The secondary outcome was overall biliary complications and 3–5 years of graft and overall survival. A meta-regression model was used to analyse the temporal trend relation in the survival outcome. The meta-analysis included 11 studies. Hazard ratios for 1-year (age cutoff of 70 and 80,) and 5-year GS (I2:0%) were similar irrespectively of the age group. The meta-regression analysis showed a significant correlation between the 1-year graft survival and the year of publication. (coef. 0.00027, 95% CI − 0.0001 to − 0.0003 *p* = 0.0009). Advanced-age donors showed an increased risk of overall biliary complications with an odd ratio (OR) of 1.89 (95% CI 1–3.65). Liver grafts potentially discharged because of high-risk failure show encouraging results, and GS in ECD has progressively improved with a temporal trend. Currently, the criteria of marginality vary amongst centres. Age alone cannot be considered amongst the extended criteria. First of all, because of the positive results in terms of septuagenarian graft survival. Moreover, the potential elderly donor-related adjunctive risk can be balanced by reducing other risk factors. A prospective multicentre study should investigate a multi-factorial model based on donor criteria, recipient features and new functional biomarkers to predict graft outcome, as proper donor–recipient matching seems to be the critical point for good outcomes.

**Supplementary Information:**

The online version contains supplementary material available at 10.1007/s13304-023-01641-1.

## Introduction

According to the last annual report from the Italian National Transplant Network, 2679 patients were on the waiting list for liver transplantation, with only 51.8% of them receiving an organ with a mortality rate on the waiting list of 4.2%, mainly due to the scarcity of donors [1]. Several strategies, including marginal donors, have been thought to increase the organ pool [2–4]. Marginal donors, also known as extended criteria donors, consist of a donor’s category whose organs could be associated with suboptimal post-transplant outcomes because of some unfavourable features. Historically, advanced donor age, viral status and steatosis grade have been concerning poor organ function and worse graft survival. Other factors considered negative prognostic factors are cold liver ischaemia time and the type of donors [5]. However, those factors may depend on logistics (organ procurement system allocation) or centre policy (definition criteria) [6]. Therefore, whether the “ideal donor” definition is relatively straightforward, there is a significant grey area where a fair potential organ may be discharged due to a lack of objective functional evaluation [7]. Nevertheless, the lack of an agreement about the marginal definition, the lack of shared criteria and their cutoff in ECD led to considerable variability of transplant outcomes for patients receiving these grafts [8, 9].

In 2016, EASL drew up guidelines that defined a non-DCD donor based on the following criteria that were recently validated: donor age > 65 years; ICU stay before donation ≥ 7 days; BMI > 30; steatosis of the liver > 40%; serum sodium > 165 mmol/L; serum transaminases > 3 times the upper limits of normal; serum bilirubin > 2 mg/dl[10]. The present study focussed on a possible correlation between advanced donor age and post-transplant survivals and complications to investigate whether age can still be considered a criterion for ECD definition.

## Materials and methods

### Search strategy

The systematic review and meta-analysis were conducted according to the PRISMA guidelines.

A computerised search of PubMed, Scopus and Cochrane Library was carried out. Reference lists of all obtained and relevant articles were screened manually and cross-referenced to identify any additional studies. Articles published from the time of inception to February 2021 were included. An advanced search was performed with the following search terms: “Grafts” or “Transplants” and “advanced age” or “older age” or “marginal donor” or “non-standard” or “extended criteria”.

### Outcomes of interest

The primary outcomes were 1-year GS with an ECD. Secondary outcomes were 3- and 5-year GS, overall biliary complication rate in organs from elderly donors, defined as biliary leakage, anastomotic and non-anastomotic strictures and hepatic arterial thrombosis.

### Inclusion criteria

Studies comparing standard donor vs. ECD reporting the primary outcome of interest were first assessed for inclusion. When two or more articles were written from the same institution or the same national database and/or author, the most recent publication was included in the analysis. Abstracts, letters, comments, editorials and expert opinions, unpublished articles and abstracts, reviews without original data and case reports were excluded from the analysis. Studies were included if considering age as a criterion of marginality, regardless of the selected cutoff. Two reviewers (I.N. and M.M.P.) independently screened the titles and abstracts of all retrieved articles. The full texts of articles that could fulfil the inclusion criteria were obtained and checked for eligibility. The following information was extracted from each article: first author, year of publication, study design, number of subjects treated, donor age, type of graft (DCD vs. DBD), ICU stay, liver donor steatosis, BMI, serum sodium, liver function tests, 1–3–5 year GS, overall biliary complications.

### Data analysis

The meta-analysis was performed using the R software suite (v3.4.0, https://www.R-project.org). The pooled effect was calculated using either the fixed-effects or the random-effects model. HR was derived from ln(HR) and SE as previously described [11]. Studies not reporting *p* value for survival analysis were excluded. An arbitrary *p* value of 0.5 was used when the studies reported the *p* as NS. Statistical heterogeneity between trials was evaluated by *χ*^2^ and *I*^2^, with significance set at *p* ≤ 0.10 [12]. In the absence of statistically significant heterogeneity, the fixed-effect method was used to combine the results. When heterogeneity was confirmed (*p* ≤ 0.10), the random-effect method was used. Potential publication bias was investigated by funnel plot. Egger’s test was used to assess funnel plot asymmetry [13], and Macaskill’s test was used to quantify the bias (14). *p* < 0.050 (two-tailed) was considered to indicate statistical significance[14]. A meta-regression model was used to explore the relationship between the year of publication, HR and age. A linear regression of HR as a function of publication year for the whole dataset was performed and contrasted with HR’s four independent linear regressions as a function of publication year stratified by age. The individual studies’ methodological quality was assessed with the CASP tool (Table 1 Supp) [15].

## Results

### Literature search

**Fig. 1:**
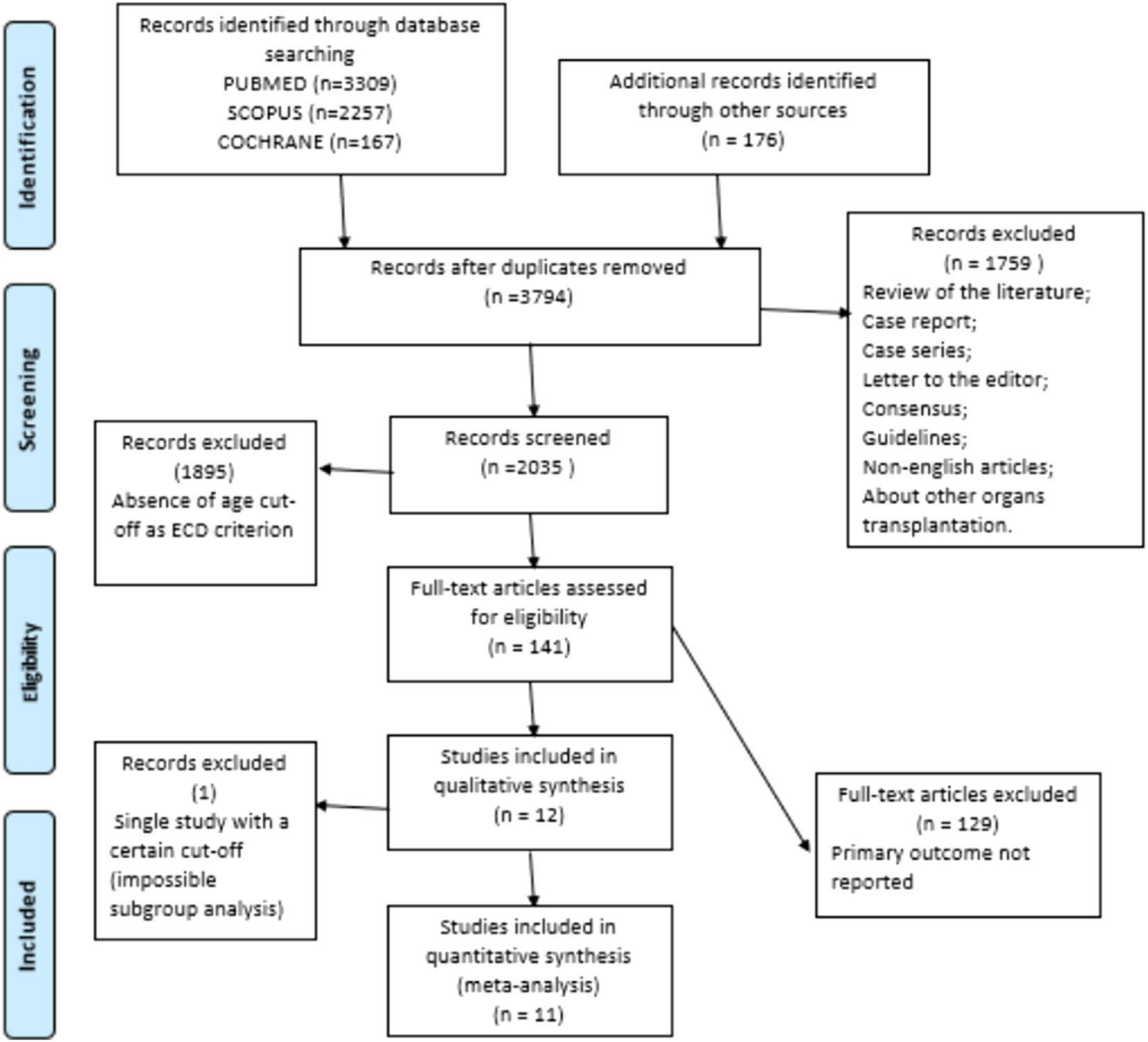
PRISMA flow diagram

The number of studies screened, assessed and excluded is reported in the PRISMA flow diagram (Fig. 1). One hundred forty-one full-text articles were assessed for eligibility, twelve were included in the qualitative analysis, whilst eleven studies fulfilled the criteria for the meta-analysis [16–27]. Confounding factors were not considered in the design of two studies, with a moderate risk of bias in the results [19, 21]. Seven studies used age as a criterion to define ECD with a cutoff [20–27], whilst five selected a discordant below 65 [16–20]. Considering other criteria presented in the recommendations, none of the selected studies considered a cutoff following the definition of ECD. In contrast, as shown in Table 1, in 29 cases, the criterion was used to define a marginal donor with a different cutoff.

**Table 1:**
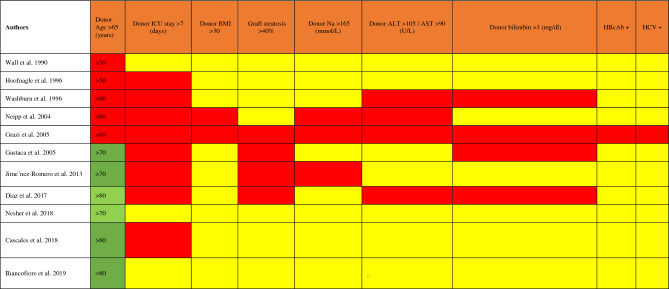
Comparison between EASL criteria for marginal liver definition (in columns) and ECD in the included papers (in rows)

Green cells indicate an accordance between the EASL cutoff and the selected paper one for the column criterion; red cells indicate that the EASL criterion was used in the selected paper to define marginality with a different cutoff; yellow cells indicate the EASL criterion was not mentioned amongst the extended criteria in the selected paper. Numbers in third column indicate the considered age cutoff for each paper

### One-year graft survival, age cutoff and temporal trend analysis

**Fig. 2:**
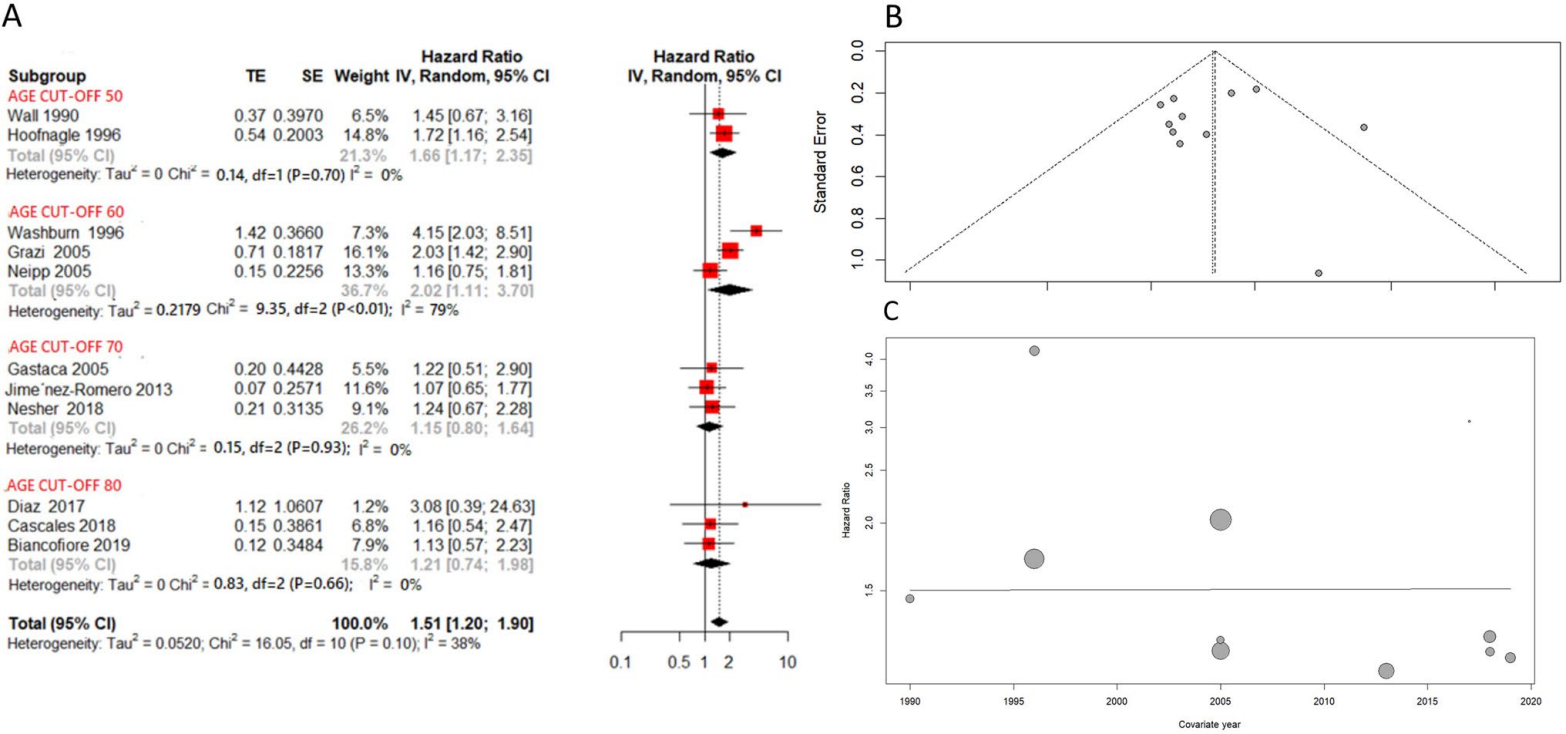
One-year graft survival, age cutoff and temporal trend subgroup analysis*.* **A** One-year GS and subgroup analysis for age cutoff (forest plot). **B** One-year GS funnel plot, **C** One-year GS bubble plot, meta-regression analysis (covariate: year)

Overall advanced-age donors showed a reduced 1-year GS compared to standard donors, with an HR of 1.51 (95% CI 1.20–1.90). The mixed-effects model (*k* = 30; tau^2 estimator: DL) tau^2 (estimated amount of residual heterogeneity) was 0.052, and *I*^2 (residual heterogeneity/unaccounted variability) was 38%. The subgroup analysis showed that older articles selected a lower age cutoff to define marginality. In the manuscripts considering 50 years (1990; 1996) and 60 years (1996; 2005) as the dividing line to mark out an elderly donor, the results in terms of 1-year graft survival were worse than in the standard donors (HR 1.66 95% CI 1.17–2.35; HR 2.02 95% CI 1.11–3.70). The most recent publications considered a higher cutoff to define a marginal donor per the EASL guidelines. Studies considering an age cutoff of 70 years (2005; 2013; 2018) or 80 years (2017; 2018; 2019) reported non-statistically significant differences in terms of post-transplant 1-year graft survival between standard and marginal donors (HR 1.15 95% CI 0.8–1.64; HR 1.21 95% CI 0.74–1.98, respectively; Fig. 2).

### Secondary outcomes

#### Three- and five-year graft survival

**Fig. 3:**
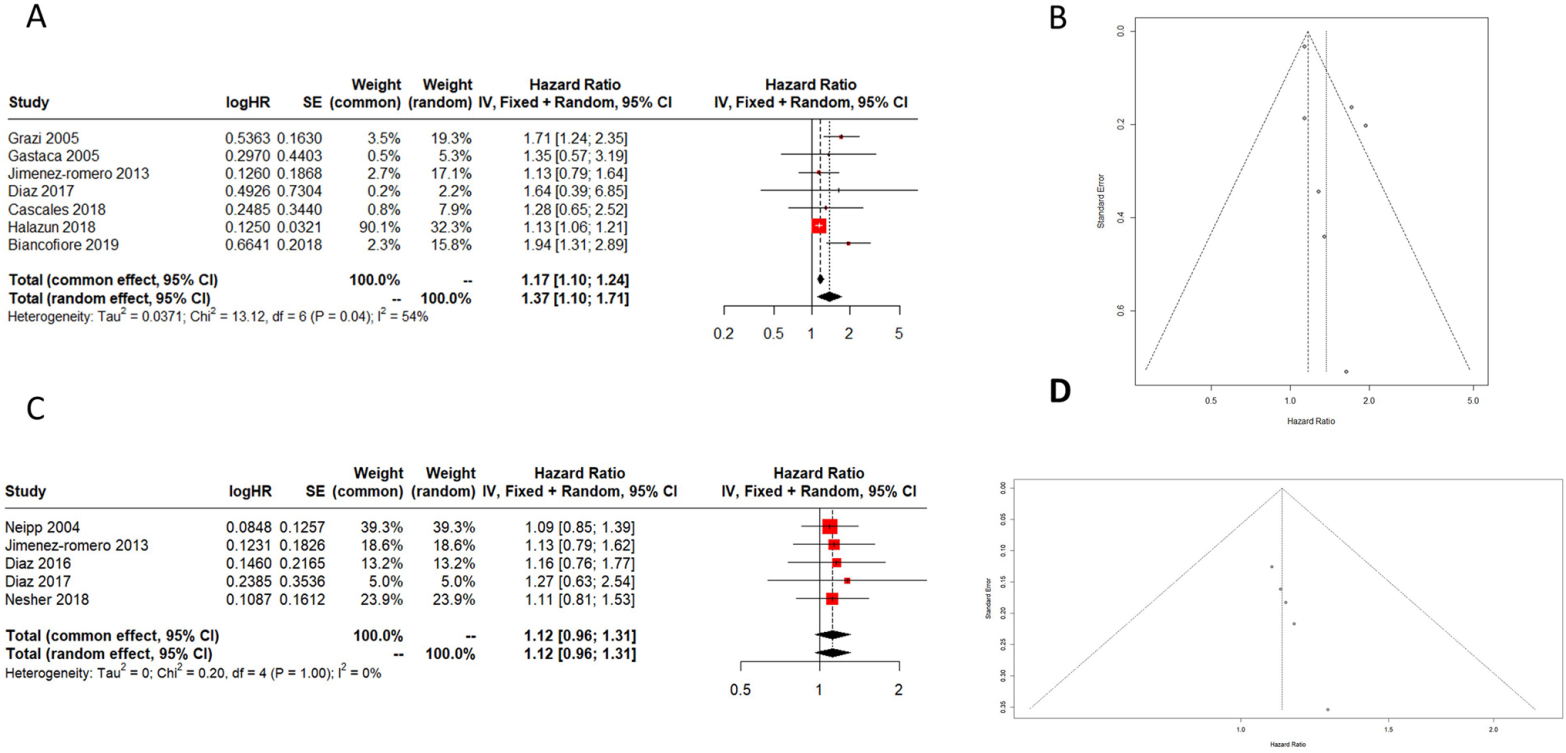
Three–five-year graft survival. **A**, **B** Forest plot and funnel plot for 3-year GS. **C**, **D** Forest plot and funnel plot for 5-year GS

Overall advanced-age donors showed a reduced 3-year GS to standard donors, with an HR of 1.37 (95% CI 1.10–1.71). The mixed-effects model (*k* = 30; tau^2 estimator: DL) tau^2 (estimated amount of residual heterogeneity) was 0.0141, and *I*^2 (residual heterogeneity/unaccounted variability) was 54% (Fig. 3A, B). 5-year GS HR of advanced-age donors compared to standard donors was 1.12 (95% CI 0.96–1.31). The mixed-effects model (*k* = 30; tau^2 estimator: DL) tau^2 (estimated amount of residual heterogeneity) was 0, and *I*^2 (residual heterogeneity/unaccounted variability) was 0% (Fig. 3C, D).

### Biliary complication and hepatic arterial thrombosis

**Fig. 4:**
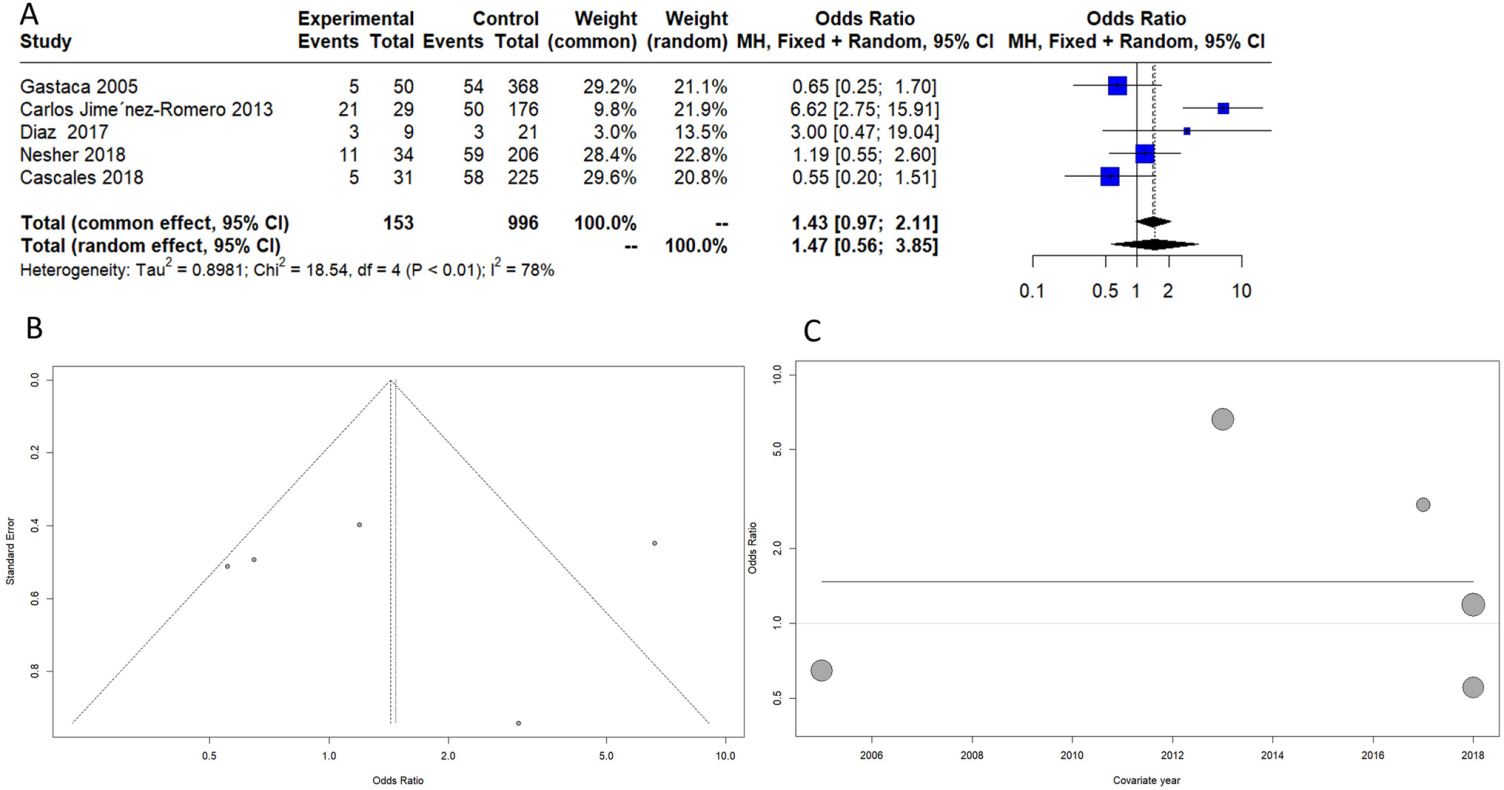
Overall biliary complications. **A** Forest plot, **B** funnel plot, **C** bubble plot

**Fig. 5:**
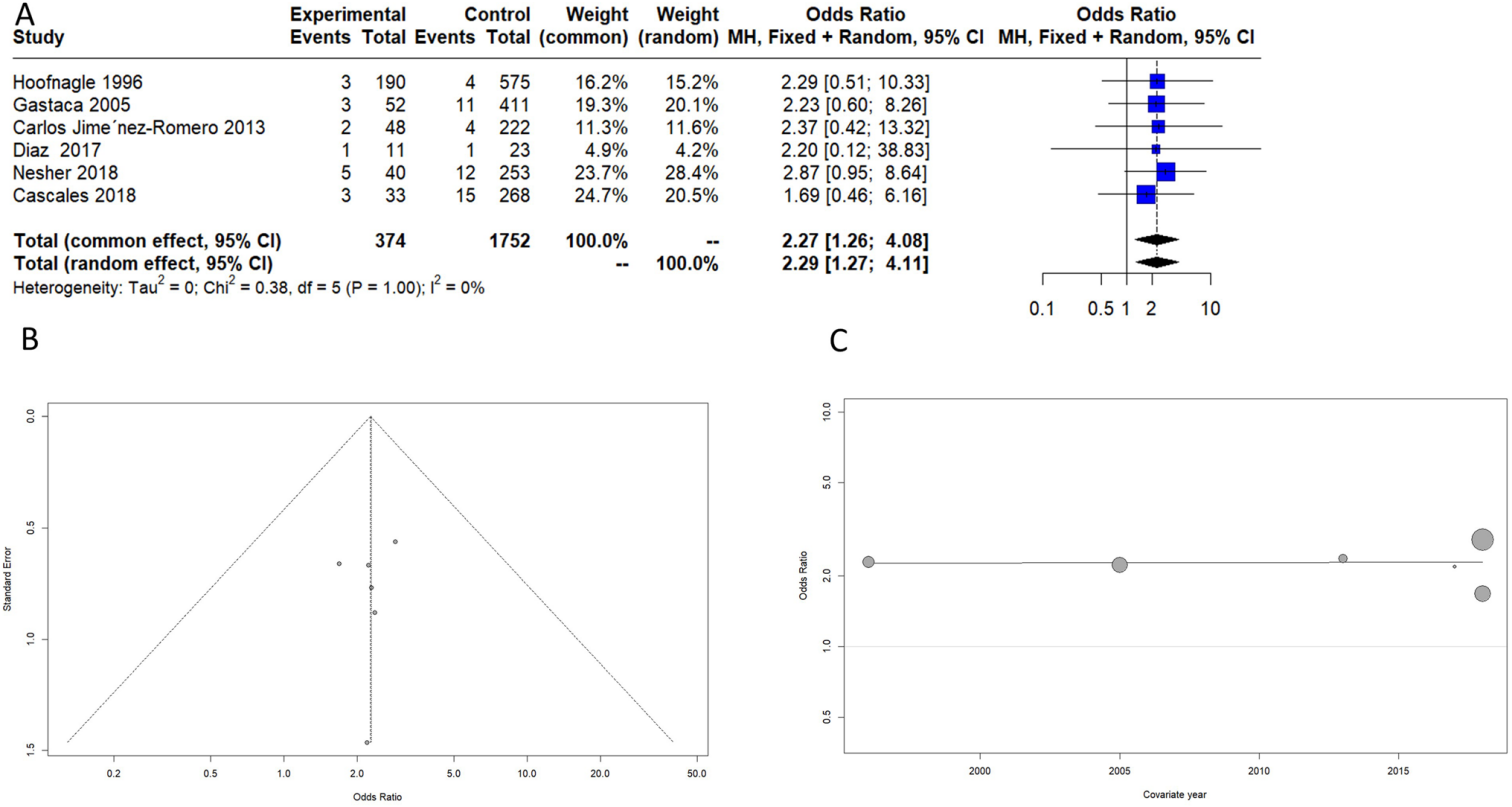
Hepatic arterial thrombosis. **A** Forest plot, **B** funnel plot, **C** bubble plot

Six selected articles reported data on overall biliary complications, as listed in Fig. 4 [21–26]. The included papers had a high grade of heterogenicity (*I*^2 = 66%) for this secondary outcome. The year of publication did not correlate with biliary complications and age. Advanced-age donors showed an increased risk of overall biliary complications with an odds ratio (OR) of 1.89 (95% CI 1–3.65). Four selected papers reported data on hepatic arterial thrombosis, as listed in Fig. 5. Advanced-age donors showed an increased risk of arterial thrombosis with an odds ratio (OR) of 2.27 (95% CI 1.26–4.08). The included papers presented converging results with an *I*^2 = 0%

## Discussion

In the last decades, the shortage of donors has led to using organs considered suboptimal or at high risk of graft loss. Although several studies have been published, the ECD donors’ definition is debated, and the weight of those criteria on graft survival is still unclear [28]. Historically, age has been considered a criterion of marginality, and most recently, DCD (marginal graft by definition) has become increasingly used in liver transplantation [7, 29]. The present study highlighted a consistent heterogeneity in the selected criteria to define a marginal donor, preventing the possibility of satisfactory outcomes comparison. [16–27]. Also, the analysis noted a significant variability of definitions within the same criterion (e.g. different age cutoffs) [16–27]. We used the EASL guidelines to classify the included manuscripts based on the ECD criteria because they are objective, only published in international guidelines and validated [10]. The present study shows that age alone cannot be considered amongst the extended criteria. First of all, because of the positive results in terms of septuagenarian graft survival and second of all because 5-year graft survival was comparable with the standard donor group.

The present analysis reported an evident correlation between the year of included article’s publication and the selected cutoff to define an elderly donor, with more recent publications using a higher age cutoff. The result probably reflects a global phenomenon of the ageing population and longer life expectancy, as well as the unstoppable organ shortage that could have pushed the interest in new resources to expand the donor pool [1]. Moreover, although a younger population was included in the ECD category, in those cases, there was a worse outcome between ECD and standard donors in terms of survival. On the other hand, considering the most recent publications, the present meta-analysis highlighted a tendency to use a higher cutoff to define an elderly donor. Nonetheless, our meta-regression presented no significant differences in terms of survival in such cases, even with elderly patients included in the ECD group. This result is undoubtedly strictly related to the recent surgical and medical progress, such as new technologies (e.g. machine perfusions) that are spreading in our clinical practise and to the steady improvement in experimental research.

The UNOS data report 2018 stressed the growth of donors over 50 and 65 years old [30]. Consequently, we may have reached the point of reconsidering the use of a fixed cutoff age to discriminate a standard from an ECD donor. Some authors recognised that age is an essential factor in graft loss, ageing, and associated comorbidities (i.e. steatosis, diabetes, and cardiovascular diseases) may influence the outcomes. Those factors should be addressed in future ECD studies [31]. Furthermore, elderly donors are more likely to be transplanted in elderly recipients with a lower graft survival influenced by recipient selection and biassed by donor-recipient matching [31].

On the other hand, even with no significant population age differences between patients receiving older or younger livers, age alone could not be considered a determinant for poorer transplantation outcomes [32]. A study highlighted that the decreased graft survival within transplant using elderly donors was limited to the graft assessed as “poor” or “fair” by the retrieval surgeon. That result suggests that the macroscopic liver evaluation correlates more than age with reduced graft survival, which may reflect additional factors not reported in the studies [17]. Unfortunately, the retrieval surgeon’s assessment is a subjective parameter, and so, by definition, it is difficult to define and standardise.

The use of ECD and elderly donors has progressively increased; to date, it represents a vital resource to increase the donor pool. However, there are several concerns regarding early complications, such as biliary complications. In this meta-analysis, we considered the overall biliary complications rate due to various data definitions from the included papers. The analysis reported discordant results amongst the included studies regarding the overall biliary complications rate. The meta-regression presented a significantly increased risk for grafts from elderly donors to undergo biliary leakage and anastomotic and non-biliary strictures. This is a non-negligible result, considering biliary complications’ impact on patients’ morbidity and mortality. The pathogenetic mechanism of biliary complications, particularly biliary strictures, is related to the extreme susceptibility of bile cells to unavoidable hypoxemic insult related to the ischaemic phase [33].

Fortunately, the modern dynamic organ preservation strategies show significant post-transplant survival results, even in grafts from DCD or high ITBL risk grafts [34]. A meta-analysis compared post-OLT complications and graft survival using HMP vs. SCS showing not only a significant decrease of biliary complications in the machine perfusion group, but also a significant increase in survival [35]. A multicentre randomised control trial comparing clinical outcomes after HOPE vs. SCS in ECD liver transplantation from DBD reported a significant decrease in post-operative serum peak ALT and AST, indicating a reduced allograft injury after reperfusion, other than a markedly reduced overall complication rate and shorter post-transplant ICU and hospital stays. [36] The implementation of machine perfusion for liver allografts represents one of the most promising efforts to improve post-operative and survival outcomes in transplantation with marginal donors and, consequently, to safely expand the donor pool. However, the present meta-analysis did not include machine perfusion studies as no comparative trials between elderly donors-machine perfusion and standard donors have been published.

One of the limitations of the present study is the unbalanced number of transplants for each group (ECD—elderly donors vs. standard). However, the results are reasonably expected as the tendency is to accept and transplant a healthy graft more than a high-risk one [36]. Therefore, a further prospective study should include the discarded organs and compare the features with the implanted ECD to analyse ECD’s actual centre-level definition. In this regard, a notable result comes from the VITTAL clinical trial. Using an NMP, most of the livers discarded by all the UK transplant centres were transplanted with a 100% 90-day overall graft survival [7]. The meta-analysis highlights the need for further studies in this direction. Another limitation of the studies is the small number of studies for every subgroup criterion, which could bias the results of the subgroup analysis. Moreover, several studies had a small sample size and potential bias at the meta-regression level.

In conclusion, the present study provides several reflections and proposals for the future. Most ECD definitions are already discussed in clinical practise and the outcomes are similar to the standard grafts. A single criterion is not enough to define an ECD. The present meta-analysis highlights the need to shift from subjective viability and quality graft assessment to a functional and dynamic evaluation. Furthermore, the paucity of comparative studies with a weak level of evidence suggests performing a large international multicentre prospective study to validate ECD criteria and draw further recommendations. The study was entirely based on published data. The authors declare that they have no conflict of interest. The data that support the findings of this study are available on request from the corresponding author F.G.

### Supplementary Information

Below is the link to the electronic supplementary material.

Supplementary file1 (DOCX 144 KB)

## Data Availability

The data that support the findings of this study are available on request from the corresponding author, F. G.

## Abbreviations

ALT: Alanine aminotransferase
AST: Aspartate aminotransferase
BMI: Body mass index
CASP: Critical appraisal skill programme
CIT: Cold ischaemia time
DBD: Donor after brain death
DCD: Donor after cardiac death
ECD: Extended criteria donor
GS: Graft survival
HMP: Hypothermic machine perfusion
HOPE: Hypothermic oxygenated machine perfusion
HR: Hazard ratio
ICU: Intensive care unit
ITBL: Ischemic type biliary lesions
NMP: Normothermic machine perfusion
NS: Non-statistically significant
PRISMA: Preferred Reporting Items for Systematic Reviews and Meta-Analyses
SE: Standard error
SCS: Static cold storage
