# Supplementary material for: Age and liver graft: a systematic review with meta-regression
Source: Updates Surg. 2023 Sep 11;75(8):2075–83. doi: 10.1007/s13304-023-01641-1 (PMC10710390; doi:10.1007/s13304-023-01641-1)
Supplement: Supplementary file 1 — Supplementary file1 (DOCX 144 KB) [file 13304_2023_1641_MOESM1_ESM.docx]

| Author | Did the study address  a clearly focused issue? | Was the cohort recruited in  an acceptable way? | Was the exposure accurately measured to  minimise bias? | Was the outcome accurately measured to  minimise bias? | Have the authors identified all  important confounding factors? | Have they taken account of the confounding factors  in the design and/or analysis? | Was the follow up of subjects complete enough? | What are the results of this study? | How precise are the results? | Do you believe the results? | Can the results be applied to the local population? | Do the results of this study fit with other available evidence? | What are the implications of this study for practice? |
| --- | --- | --- | --- | --- | --- | --- | --- | --- | --- | --- | --- | --- | --- |
| Wall et al. 1990 | Yes | Can't Tell | Can't Tell | Yes | Yes | Yes | Yes | Results quite clear; good association between exposure and outcome | Good precision | Yes | Yes | Can't Tell | The paper is old |
| Hoofnagle et al. 1996 | Yes | Can't Tell | Yes | Yes | Can't Tell | Can't Tell | Yes | An older donor liver adversely affected outcome of liver transplantation | Good precision | Yes | Yes | Yes | Older donors were correlated with worse outcomes |
| Washburn et al. 1996 | Yes | Yes | Yes | Can't Tell | Can't Tell | No | Yes | Cautious use of older liver grafts in healthier recipients | Good precision | Yes | Can't Tell | Yes | Good evidence |
| Neipp et al. 2004 | Yes | Yes | Can't Tell | Yes | Yes | No | Yes | The outcome of LTx with over 60 graft is comparable to results of LTx with under 60 graft | Good precision | Yes | Yes | Yes | Good evidence |
| Grazi et al. 2005 | Yes | Can't Tell | Can't Tell | Yes | Can't Tell | Can't Tell | Can't Tell | The graft survival rate of older donors is lower than that of younger donors | Good precision | Yes | Can't Tell | Yes | Good evidence for that time |
| Gastaca et al. 2005 | yes | yes | Can't Tell | Can't Tell | Can't Tell | Can't Tell | yes | for donor >70 years old the outcomes were comparable to those obtained with younger donors. | Good precision | yes | yes | yes | Strong evidences |
| Jiménez-Romero C. et al 2013 | yes | yes | Can't Tell | Can't Tell | Can't Tell | Can't Tell | Yes | no difrences in survival between elderly and younger donors | Good precision | yes | yes | Yes | used of elderly donors |
| Diaz et al 2017 | yes | yes | yes | yes | yes | yes | yes | Livers from older donors can be safely used for transplantation with acceptable patient survival rates, but with lower survival without retransplant | Good precision | yes | yes | yes | good evidence |
| Cascales et al. 2018 | yes | yes | Can't Tell | Can't Tell | Can't Tell | Can't Tell | yes | The use of octogenarian donors makes it possible to increase the pool of donors while providing enough safety for the recipient. | Good precision | yes | yes | yes | Strong evidences |
| Nesher et al. 2018 | Yes | Can't Tell | Can't Tell | Yes | Yes | Can't Tell | Yes | Grafts from donors older than 70 years may be equally safe if cold ischemia is maintained for less than 8 hours | Good precision | Yes | Yes | Yes | Quite weak |
| Biancofiore et al. 2019 | yes | yes | Can't Tell | Can't Tell | yes | yes | yes | Octogenarian donors in liver transplantation grant an equivalent perioperative course to ideal young donors. | Good precision | yes | yes | yes | Strong evidences |

**Table 1 Supp. CASP checklist**
